# Luteoma of Pregnancy Presenting as Ruptured Ectopic Pregnancy: A Case Report

**DOI:** 10.7759/cureus.30900

**Published:** 2022-10-31

**Authors:** Ishita Agarwal, Jasmina Begum, Naimisha P Singupuram

**Affiliations:** 1 Obstetrics and Gynecology, All India Institute of Medical Sciences, Bhubaneswar, IND

**Keywords:** adnexal masses, ovarian neoplasms, ectopic, pregnancy, luteoma

## Abstract

Luteoma of pregnancy is a rare, benign neoplasm arising from the ovary, which occurs due to pregnancy-induced hormones.This rare ovarian lesion was first described by Sternberg and Barclay in 1966. Our case is unusual as the ovarian mass was misdiagnosed as ruptured ectopic pregnancy. Only three such cases have been previously reported in the literature.

A 28-year-old multigravida with three months of amenorrhea presented with vaginal bleeding, abdominal pain, and gradually increasing vertigo for six days with increased intensity in the last four hours. On examination, she was conscious and oriented, clinically moderate pallor was present, her pulse rate was 112 beats per minute (bpm), and her blood pressure (BP) was 98/68 mm Hg. On abdominal examination, there was no palpable abdominal mass, but left iliac fossa guarding and tenderness were present. On per-vaginal examination, the uterus was eight weeks in size, the right fornix was free, the left fornix was full and tender, and cervical motion tenderness was present. Her urine pregnancy test was positive. Transvaginal sonography was performed in the emergency setting, which showed a bulky uterus with thickened endometrium and a non-visualized right ovary, and the left ovary was seen adjacent to a hyperechoic collection in the pouch of Douglas of size 3.5×3.5×1.8 cm, likely organized hematoma; there was free fluid in the pouch of Douglas, and left forniceal tenderness was also present. In view of the clinical evidence of tachycardia and hypotension, an exploratory laparotomy was performed for suspected ruptured ectopic pregnancy, and the ovarian mass was excised. The histopathological examination (HPE) of the ovarian mass showed findings suggestive of luteoma of pregnancy.

There is an extreme paucity of literature on luteoma of pregnancy. That, along with the rarity of the lesion, results in it often not being kept in mind as a differential diagnosis on clinical or radiological examination, thereby leading to more aggressive management. Obstetricians and gynecologists need to be aware of this condition so that it is kept as a differential diagnosis in patients presenting with adnexal masses. A vigilant outlook will help in preventing unnecessary radical surgery during pregnancy, thereby preserving the ovary and reducing morbidity in these young females.

## Introduction

Luteoma of pregnancy is a benign ovarian neoplasm that occurs due to pregnancy-induced hormones [1]. This rare ovarian lesion was first described by Sternberg and Barclay in 1966 [2]. Luteoma has been found mostly associated with maternal or fetal virilization [3-5]. Most commonly, it is found incidentally on imaging or during cesarean delivery/postpartum tubal ligation [2]. Large luteomas have been found associated with complications caused by their mass effect [6]. Our case is unusual as the ovarian mass was misdiagnosed as a case of ruptured ectopic pregnancy. Only three such cases have been previously reported in the literature based on bibliographical research [3,6,7].

## Case presentation

A 28-year-old multigravida with two previous living issues with no significant past medical or family history presented to the gynecological emergency with a history of three months of amenorrhea with chief complaints of vaginal bleeding, abdominal pain, and gradually increasing vertigo for six days with increased intensity in the last four hours. The patient revealed that this pregnancy had been conceived spontaneously. The patient had been referred from a primary healthcare center with features suspicious of chronic ectopic pregnancy.

On examination, she was conscious and oriented, clinically moderate pallor was present, her pulse rate was 112 beats per minute (bpm), and her blood pressure (BP) was 98/68 mm Hg. On abdominal examination, there was no palpable abdominal mass, but left iliac fossa guarding and tenderness were present. On per-vaginal examination, the uterus was eight weeks in size, the right fornix was free, the left fornix was full and tender, and cervical motion tenderness was present. The rest of the examination findings were normal with no features of hirsutism or virilization.

**Figure 1:**
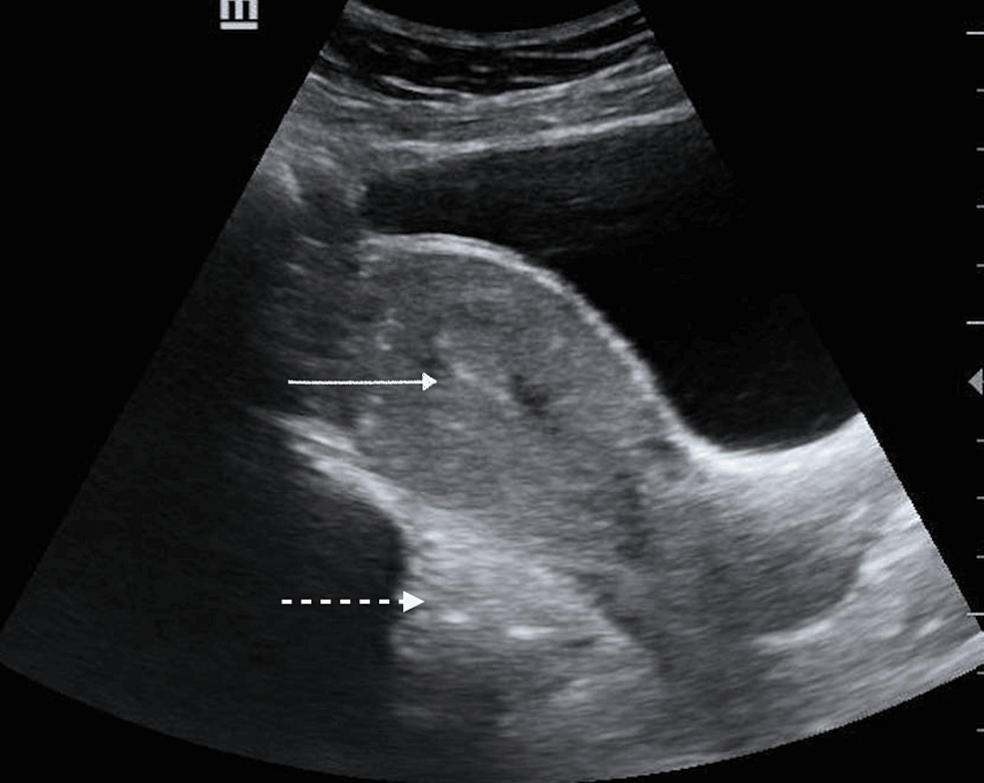
Transvaginal ultrasound image Thickened endometrium with no intrauterine gestational sac (solid white arrow) with hyperechoic collection in the pouch of Douglas (dotted white arrow)

Her urine pregnancy test was positive. Transvaginal ultrasonography was performed in the emergency setting, which showed a bulky uterus with thickened endometrium and a non-visualized right ovary, and the left ovary was seen adjacent to a hyperechoic collection in the pouch of Douglas of size 3.5×3.5×1.8 cm, likely organized hematoma; there was free fluid in the pouch of Douglas, and left forniceal probe tenderness was also present (Figure 1).

Based on the abovementioned findings, a provisional diagnosis of ruptured ectopic pregnancy was made. Her preoperative blood investigations revealed a hemoglobin value of 7.3 g/dl and serum beta human chorionic gonadotropin (hCG) value of 5150 mIU/ml. The patient was planned for emergency laparotomy (in view of the clinical evidence of tachycardia and hypotension) after matching blood and plasma products for potential transfusion.

**Figure 2:**
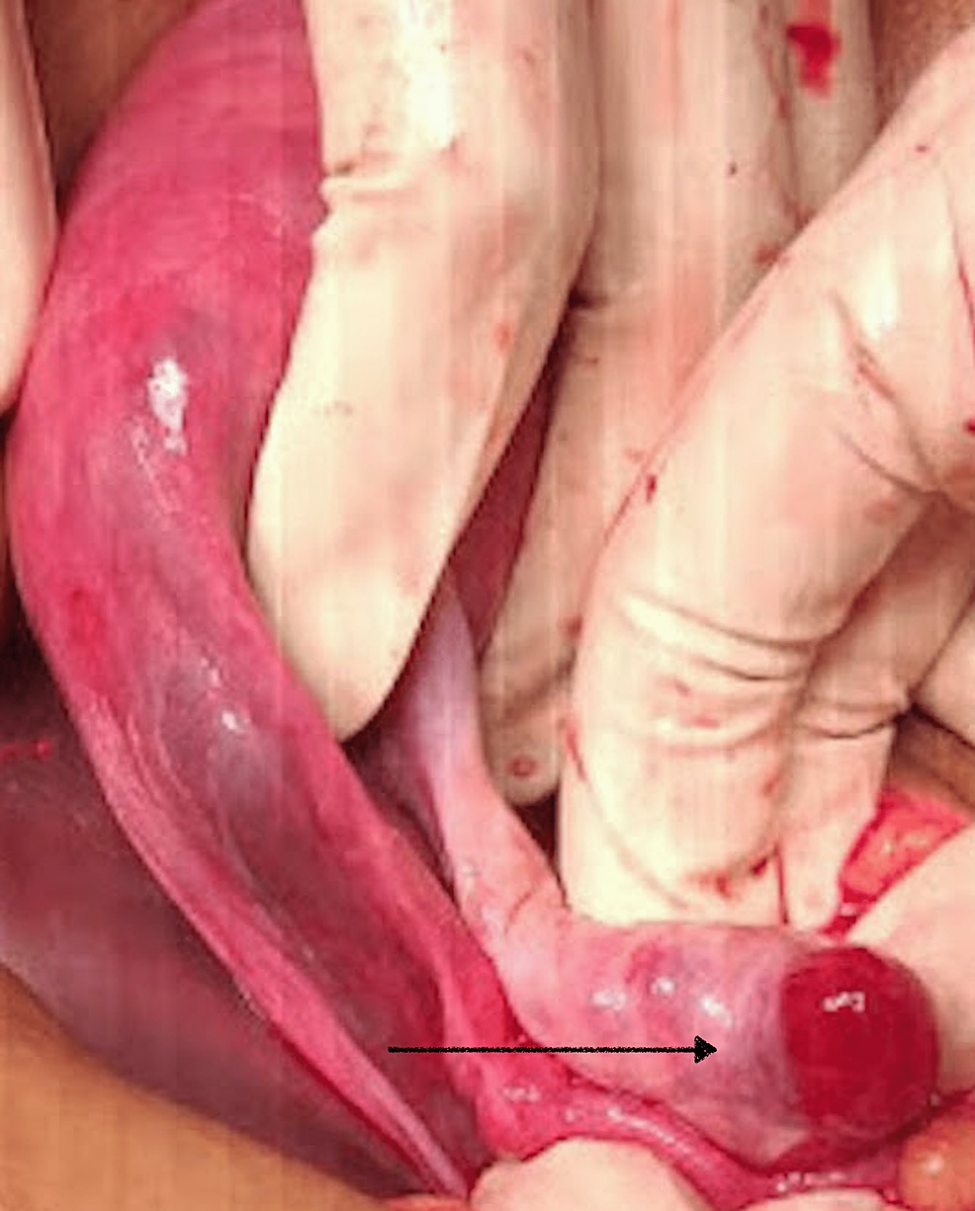
Intraoperative image A 2×2 cm well-circumscribed mass arising from the left ovary (black arrow)

Emergency laparotomy was performed. On opening the abdomen, hemoperitoneum was seen. Approximately 100 ml of clotted hematoma and 150 ml of fresh unclotted blood were suctioned out; a 2×2 cm well-circumscribed mass was seen arising from the left ovary (Figure 2).

**Figure 3:**
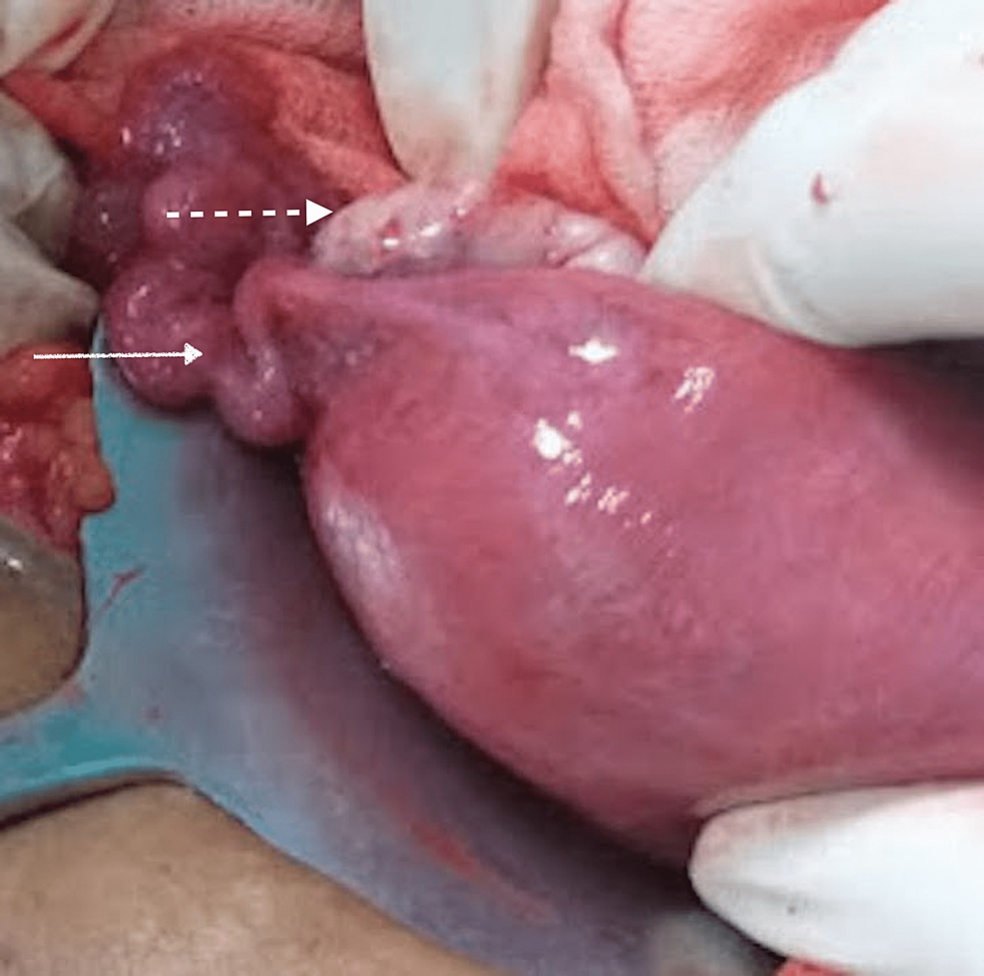
Intraoperative image Normal right fallopian tube (solid arrow) and ovary (dotted arrow)

Adhesions were present between the left fallopian tube and the left ovary; the right ovary and the right fallopian tube were normal (Figure 3).

**Figure 4:**
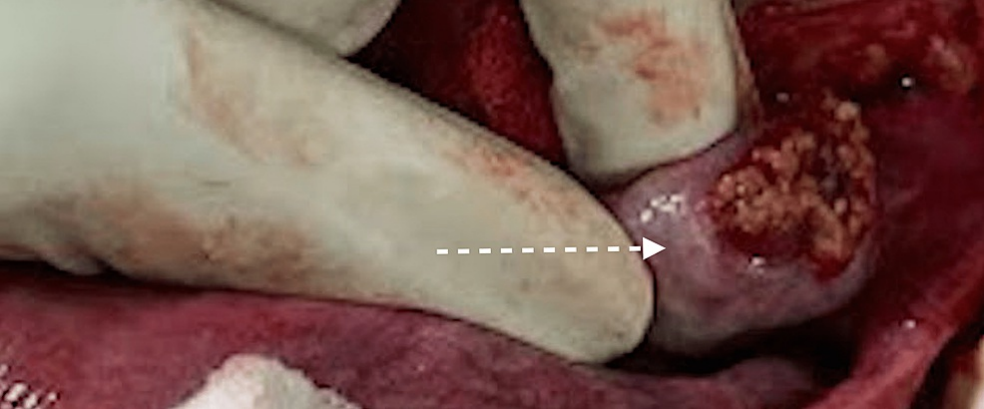
Intraoperative image The cut section of the mass showing yellow whorled fleshy areas with focal areas of hemorrhage (dotted arrow)

The cut section of the nodular mass revealed yellow whorled fleshy areas with focal areas of hemorrhage (Figure 4).

**Figure 5:**
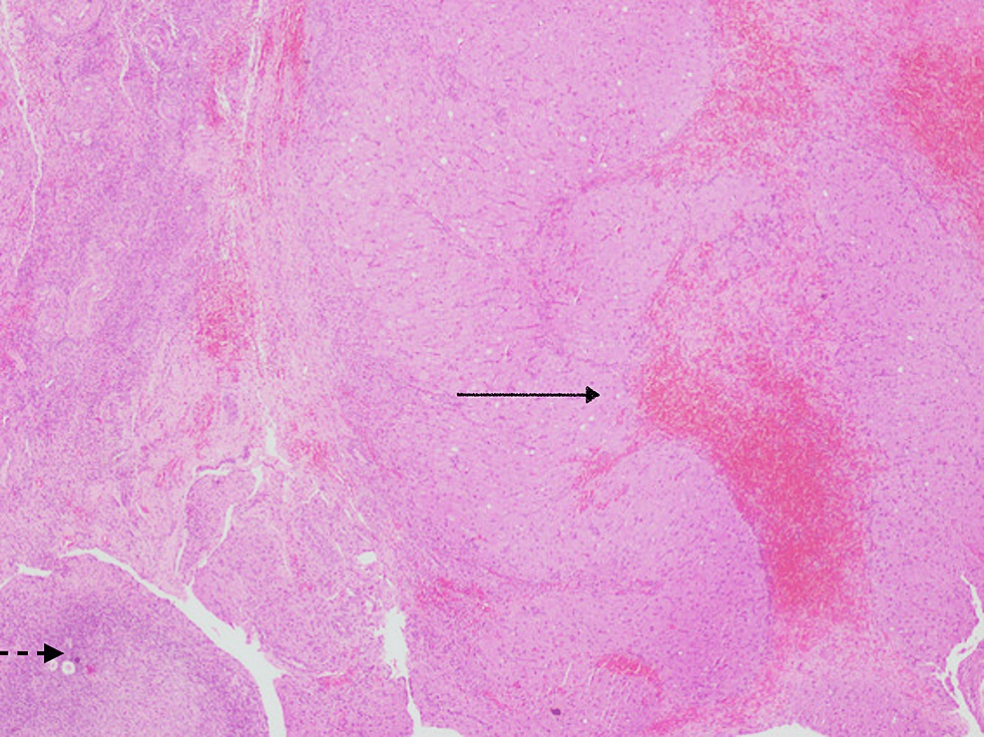
Histopathology image Ovarian tissue (dotted arrow) with large polygonal cells with abundant eosinophilic cytoplasm and central nucleus (solid arrow), suggestive of luteoma of pregnancy

**Figure 6:**
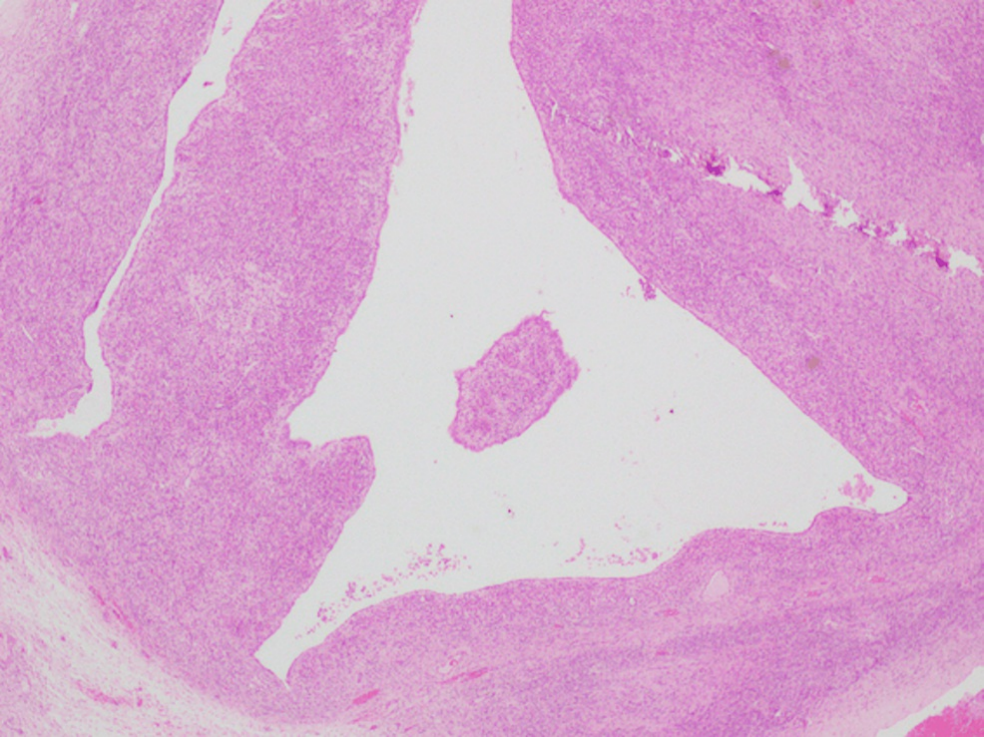
Histopathology image A follicular cyst with extensive areas of luteinization

The fleshy mass was excised preserving the normal ovarian tissue, and the mass was sent for histopathological examination (HPE). She was transfused with one unit of packed red blood cells (PRBC) intraoperatively. In view of the intraoperative findings that were unusual for ruptured ectopic pregnancy, a suspicious diagnosis of ruptured corpus luteum cyst was made. Dilation and curettage of the endometrial cavity was done. Moderate amount of residual products of conception was curetted out and sent for HPE. On HPE, paraffin-embedded sections showed ovarian tissue with large polygonal cells with abundant eosinophilic cytoplasm and central nucleus, features suggestive of luteoma of pregnancy (Figure 5), along with a follicular cyst with extensive areas of luteinization (Figure 6).

The curettage sample showed evidence of residual products of conception with scant chorionic villi. Both the samples showed no evidence of atypia, mitosis, or any other features suggestive of malignancy. Based on the abovementioned clinical and pathological findings, a final diagnosis of missed abortion with luteoma of pregnancy was made. Her postoperative hemoglobin level was 8 g/dl (after one unit of PRBC transfusion). Her postoperative period was uneventful, and she was discharged in stable condition after four days and asked to follow up in the gynecology outpatient service. Her urine pregnancy test done at two weeks of follow-up visit came negative, suggestive of normal beta hCG levels.

## Discussion

Luteoma of pregnancy is said to occur as a result of theca cell proliferation under the effect of increased levels of beta hCG in pregnancy [2]. There can be a variety of differential diagnoses for luteoma of pregnancy, ranging from benign to malignant. Corpus luteoma of pregnancy is an important differential diagnosis as an ovarian mass seen in early pregnancy [1], but it shows a histopathological architecture of convoluted folds arranged around a fibrovascular center forming its core [5]. Another common benign differential is hyperreactio luteinalis, but it is more cystic and mostly involves bilateral ovaries [1].

Among the malignant ovarian masses, steroid cell tumors form a very close differential, in that they also present with features of virilization, but they have a distinct vascular structure on microscopic examination, which helps in differentiation [1]. Luteinized granulosa cell tumors and thecomas have non-luteinized cells on microscopy [5].

Luteoma of pregnancy should be suspected in patients with underlying polycystic ovarian syndrome (PCOS), as PCOS predisposes to increased beta hCG levels, thus increasing the risk of the formation of luteoma of pregnancy [4,8]. These tumors rarely present in early pregnancy [5]. As these lesions are benign and regress spontaneously after pregnancy once the pregnancy-associated hormonal changes revert back, only patients with virilizing symptoms or complications such as torsion require immediate surgery [5]. Our patient did not have any features of virilization or torsion but warranted surgery as it was a case of ruptured luteoma of pregnancy. In general, conservative management should be done for asymptomatic cases, which only require regular follow-up post delivery [9]. In cases having atypical presentations, frozen section examination can be performed intraoperatively, which will help in preserving the ovary and the fallopian tube of the young female [10].

## Conclusions

As luteoma of pregnancy is a rare lesion, it is often not kept in mind on clinical or radiological examination, thereby leading to a misleading diagnosis and more aggressive management. Obstetricians and gynecologists need to be aware of this condition so that it is kept as a differential diagnosis in patients presenting with adnexal masses. A vigilant outlook will help in preventing unnecessary radical surgery during pregnancy, thereby reducing morbidity in these young females.
